# Overview of the prevalence of job satisfaction and turnover intention among emergency medical services personnel: a meta-analysis

**DOI:** 10.7189/jogh.15.04320

**Published:** 2025-11-28

**Authors:** Garry Huang, Wei-Kang Hung, Reymond Ngolombe, Christopher Maona, Burnett Chila Chiona, Kondwani Joseph Banda

**Affiliations:** 1 Taipei Medical University Shuang Ho Campus, School of Health Care Administration, New Taipei City, Taiwan; 2 Australasian College of Paramedicine, Sydney, Australia; 3 National Taiwan Normal University, Department of Civic Education and Leadership, Taipei, Taiwan; 4 National Taipei University of Nursing and Health Sciences, School of Nursing, Taipei, Taiwan; 5 Mzimba District Hospital, Department of Nursing, Mzimba, Malawi; 6 Ekwendeni College of Health Sciences, Department of Medical Surgical Nursing, Mzimba, Malawi; 7 Taipei Medical University, International PhD Program in Gerontology and Long-Term Care, Taipei, Taiwan; 8 St John’s Institute for Health, Department of Medical Surgical Nursing, Mzuzu, Malawi; 9 Taipei Medical University, School of Nursing, Taipei, Taiwan; 10 Kamuzu Central Hospital, Department of Surgery, Lilongwe, Malawi

## Abstract

**Background:**

Emergency medical services (EMS) personnel, including paramedics, emergency medical technicians (EMTs), and firefighters are subjected to substantial occupational stressors that diminish job satisfaction and increase turnover rate, ultimately affecting efficient delivery of pre-hospital emergency care. Therefore, we performed the first meta-analysis to determine the prevalence of job satisfaction and turnover intention among EMS personnel, including paramedics, emergency medical technicians (EMTs), and firefighters.

**Methods:**

We comprehensively searched Web of Science, PubMed, Cochrane Library, Embase, and EBSCOhost until March 2025. The pooled prevalence of job satisfaction and turnover intention was analysed using the Freeman-Tukey double-arcsine transformation model in R software. Cochran's Q and statistics assessed heterogeneity and subgroup analysis explored moderator variables.

**Results:**

A total of 25 studies with 59 562 EMS personnel were included. The pooled prevalence of job satisfaction was 63% (95% confidence interval (CI) = 53%, 72%), with estimates of 71% for EMTs, 62% for firefighters, and 54% for paramedics. Job satisfaction was 56% during the COVID-19 pandemic and 65% in the pre-pandemic period. The pooled prevalence of turnover intention was 29% (95% CI = 24%, 36%), with estimates of 28% for paramedics, 22% for EMTs, and 17% for firefighters. Turnover intention was 34% during COVID-19 pandemic and 27% in the pre-pandemic period.

**Conclusions:**

Approximately, 63% of EMS personnel report job satisfaction, while 29% express intent to leave the profession. Mental health support, workload management, and professional development opportunities should be promoted among EMS personnel to further enhance job satisfaction and mitigate turnover intention.

**Registration:**

PROSPERO: CRD420251027283.

Globally, Emergency Medical Services (EMS) personnel, including paramedics, emergency medical technicians (EMTs), and firefighters play a crucial role in providing pre-hospital urgent and life-saving care to individuals [1,2]. Emergency Medical Services personnel face significant occupational stressors, negatively impacting job satisfaction, including high psychological stress, physical exertion, and exposure to traumatic events [3,4]. Premature attrition from EMS has been shown to be attributed to the adverse effects of rotating night shifts, substantial cognitive and physical fatigue, workplace conflict, and occupational injury or violence [5], further contributing to reduced job satisfaction. Moreover, the demanding nature of the pre-hospital emergency services is further compounded by inflexible work schedules, shift work, and mandatory overtime, exacerbating the physical and psychological burden on EMS personnel and further reducing their job satisfaction [2,5]. Therefore, identifying the extent of job satisfaction and turnover intention among EMS personnel is essential for developing targeted strategies to enhance satisfaction and reduce turnover.

Previous research has identified key factors contributing to decreased job satisfaction and turnover intention among EMS personnel [6–9], including excessive workloads, irregular and prolonged shift schedules, exposure to traumatic events, and high risk of workplace violence. Additionally, inadequate organisational support, limited opportunities for career advancement, low compensation, and poor work-life balance further reduce job morale [5,10]. These challenges are often exacerbated by insufficient medical supplies, understaffing, and lack of access to mental health services creating a work environment that fosters job dissatisfaction and drives intention to leave the profession among EMS personnel. Moreover, previous findings have emphasised the ongoing need to address mental health and well-being of EMS personnel, which are strongly associated with job satisfaction, a well-established predictor of turnover among health care workers [4–6]. Furthermore, EMS managers play a critical role in providing organisational support and training to enhance performance and thus, improve job satisfaction among EMS personnel [8,9]. However, evidence on the extent and prevalence of job satisfaction and turnover intention among EMS personnel remain unknown as previous studies have predominantly focused on physicians and nurses. Thus, owing to significant physical and mental challenges within the EMS field, further investigation into the prevalence of job satisfaction and turnover intention among EMS personnel is necessary to inform policy and develop effective job satisfaction and retention interventions.

Given the essential role of EMS personnel, including paramedics, emergency medical technicians (EMTs), and firefighters in health care delivery and the demanding nature of their work can impede and prevent effective delivery of pre-hospital emergency services. As such, understanding the current status of job satisfaction and turnover intention among EMS personnel is warranted. Therefore, we conducted the first meta-analysis to determine the prevalence of job satisfaction and turnover intention among EMS personnel.

## METHODS

### Search strategy

This study was registered in PROSPERO: CRD420251027283 and Meta-Analysis of Observational Studies in Epidemiology (MOOSE) guidelines was used in reporting the current meta-analysis [11,12]. A comprehensive search from inception of each database was conducted in Web of Science, PubMed, Cochrane Library, Embase, and EBSCOhost was first conducted in October 2024 with a subsequent search in March 2025. The following keywords were used in combination with a detailed search strategy in the Table S1 in the **Online Supplementary Document**: (prevalence OR incidence OR epidemiology OR rate OR rates OR number OR proportion OR probability OR event) AND (job satisfaction OR intrinsic satisfaction OR extrinsic satisfaction OR turnover intention OR intention to leave OR attrition) AND Emergency Medical Services Personnel OR EMS personnel OR ambulance personnel OR fire fighters OR first responders OR paramedics OR emergency medical technicians OR EMTs). Google search was performed for studies that were identified through manual search in relevant previous observational studies, systematic review, and meta-analysis to identify other potential studies. Additionally, original authors were contacted through emails when there was missing data in the published studies to include all eligible studies, and the studies were excluded if no response at the time of the analysis.

### Study selection

The inclusion criteria for included studies without any language restrictions was as follows:

(1) population: EMS personnel, including paramedics, emergency medical technicians (EMTs), and firefighters;

(2) exposure of interest: job satisfaction or turnover intention;

(3) comparison: no job satisfaction or turnover intention;

(4) outcome of interest: prevalence;

(5) study design: observational studies including cross-sectional and prospective studies.

The exclusion criteria were as follows:

(1) duplicate studies,

(2) non-relevant population studies,

(3) non-observational studies,

(4) systematic review or meta-analysis studies,

(5) studies unrelated to the topic.

### Data extraction

GH and KJB, as independent reviewers, performed the data extraction using standardised forms and tables designed by the authors in Microsoft Word and Microsoft Excel 2021 (Version 2108, Microsoft Corporation), with the following categories; author, year of publication, age, sample size, gender, study design, country, continent, EMS personnel (paramedics, EMTs, firefighters, and mixed personnel), assessment method, and outcome (job satisfaction and turnover intention) in the included studies. Notably, the mixed EMS personnel group comprised of studies that reported the prevalence of job satisfaction and turnover intention for all EMS personnel, including paramedics, EMTs, and firefighters, without providing separate estimates for each group. The study outcomes were pooled prevalence of job satisfaction and turnover intention among EMS personnel (**Table 1**).

**Table 1:** Study characteristics for included studies

Author (year)	Country, continent	Age (x̄ ± SD)	Sample (n), gender (M/F/NA)	Study design	EMS personnel	COVID-19 Status	Study outcome (tool), prevalence (n and %)	Study quality
Al-Mansour (2021) [13]	Saudi Arabia, Asia	38.9 ± 8.1	n = 310, gender = NA	Cross-sectional	Paramedics	During	Turnover intention (SI), 84 (27.1)	9 – low risk
Baier et al. (2018) [14]	Germany, Europe	≥18	n = 1101, gender = 949/152	Cross-sectional	Mixed	Before	Job satisfaction (SI), 591 (53.7)	7 – moderate risk
							Turnover intention (SI), 601 (54.6)	
Beaton & Murphy (1993) [15]	USA, North America	37.2 ± 7.5	n = 1750, Gender = 1610/140	Retrospective	Mixed	Before	Job satisfaction (SI), 1192 (68.1)	7 – moderate risk
Blau et al. (2009) [16]	USA, North America	≥18	n = 854, gender = NA	Cross-sectional	Mixed	Before	Job satisfaction (SI), 692 (81.0)	8 – moderate risk
							Turnover intention (SI), 273 (32.0)	
Blau et al. (2016) [17]	USA, North America	≥18	n = 2815, gender = NA	Retrospective	Paramedics	Before	Job satisfaction (SI), 493 (60.0)	7 – moderate risk
							Turnover intention (SI), 287 (34.9)	
					EMTs	Before	Job satisfaction (SI), 397 (75.0)	
							Turnover intention (SI), 185 (35.0)	
Bowron & Todd, (1999) [18]	USA, North America	32.3	n = 90, gender = 51/39	Cross-sectional	Mixed	Before	Job satisfaction (SI), 77 (85.6)	7 – moderate risk
Cash et al. (2019) [19]	USA, North America	38.3 (3.2)	n = 2815, gender: 1700/735	Cross-sectional	Mixed	Before	Job satisfaction (SI), 2275 (80.8)	7–Moderate Risk
							Turnover intention (SI), 839 (29.8)	
Chapman et al. (2009) [20]	USA, North America	≥18	n = 933, gender = 662/271	Cross-sectional	Paramedics	Before	Job satisfaction (SQ), intrinsic = 541 (86.5), extrinsic = 455 (72.8)	8 – moderate risk
							Turnover intention (SI), 209 (33.4)	
					EMTs	Before	Job satisfaction (SQ), intrinsic = 265 (86.0), extrinsic = 234 (76.0)	8 – moderate risk
							Turnover intention (SI), 99 (32.2)	
Chapman et al. (2016) [21]	USA, North America	≥18	n = 4202, gender = NA	Retrospective	Paramedics	Before	Job satisfaction (SQ), 802 (91.6)	7 – moderate risk
							Turnover intention (SI), 66 (8.0)	
					EMTs	Before	Job satisfaction (SQ), 3189 (95.6)	
							Turnover intention (SI), 217 (7.0)	
Chegini et al. (2022) [22]	Iran, Asia	33.1 ± 6.8	n = 198, gender = 167/31	Cross-sectional	Mixed	During	Job satisfaction (MSQ), 101 (51.0)	9 – low risk
Crowe et al. (2018) [23]	USA, North America	≥18	n = 2153, gender = 1588/565	Cross-sectional	Paramedics	Before	Job satisfaction (SI), 115 (8.0)	9 – low risk
							Turnover Intention (SI), 271 (19.7)	
					EMTs	Before	Job satisfaction (SI), 59 (8.0)	
							Turnover intention (SI), 182 (23.4)	
Eiche et al. (2021) [24]	Germany, Europe	≥18	n = 2590, gender = 2079/508	Cross-sectional	Paramedics	Before	Job satisfaction (JSQ), 1684 (65.0)	9 – low risk
							Turnover intention (SI), 1399 (54.0)	
Hulkkonen et al. (2024) [25]	Finland, Europe	≥18	n = 433, gender = 247/177/9	Cross-sectional	Paramedics	During	Turnover intention (SI), 121 (27.9)	8 – moderate risk
Hwang & Chang (2008) [26]	South Korea, Asia	≥18	n = 214 gender = 87/127	Retrospective	Paramedics	Before	Turnover intention (SI), 171 (79.9)	8 – moderate risk
Jahnke et al. (2019) [27]	USA, North America	40.2 ± 9.0	n = 1773, gender = NA	Retrospective	Firefighters	Before	Job satisfaction (SI), 1355 (76.4)	7 – moderate risk
Muthukumaran et al. (2023) [28]	Malaysia, Asia	38.7 (9.0)	n = 6041, gender: 5794/247	Cross-sectional	Firefighters	Before	Job satisfaction (SSI), 4235 (70.1)	9 – low risk
Nirel et al. (2008) [29]	Israel, Asia	≥18	n = 328, gender = 286/42	Cross-sectional	Paramedics	Before	Job satisfaction (SQ), 197 (60.1)	8 – moderate risk
							Turnover intention (IQ), 52 (15.9)	
North et al. (2002) [30]	USA, North America	≥18	n = 181, gender = 176/5	Cross-sectional	Firefighters	Before	Job satisfaction (DS), 72 (39.8)	7 – moderate risk
							Turnover intention (DS), 22 (12.1)	
Patterson et al. (2010) [31]	USA, North America	≥18	n = 1595, gender = 1141/454	Cross-sectional	Mixed	Before	Job satisfaction (EMS-SAQ), 1101 (69.0)	9 – low risk
							Turnover Intention (EMS-SAQ), 638 (40.0)	
Perkins et al. (2009) [32]	USA, North America	35.0 (NA)	n = 1008, gender = NA	Cross-sectional	EMTs	Before	Job satisfaction (SDQ), 917 (91.0)	9 – low risk
							Turnover intention (SDQ), 242 (24.0)	
Raposa et al. (2023) [32]	USA, North America	39.5 ± 9.8, n = 5467	n = 5467, gender = 4971/362/135	Cross-sectional	Mixed (FOCUS)	During	Job satisfaction (SDQ), 3007 (55.0)	9 – low risk
							Turnover intention (SDQ), 2187 (40.0)	
		38.5 ± 8.5	n = 2172, gender = 1859/300/13	Cross-Sectional	Mixed (SAVER)	During	Job satisfaction (SDQ), 1086 (50.0)	
							Turnover intention (SDQ), 912 (42.0)	
Rivard et al. (2020) [33]	USA, North America	38.5 ± 3.0	n = 18 284, gender = 13 600/4643/41	Retrospective	Mixed	Before	Job satisfaction (SDQ), 16 913 (92.5)	8 – moderate risk
							Turnover intention (SDQ), 4742 (25.9)	
Saijo et al. (2008) [34]	Japan, Asia	≥18	n = 1301, gender: 1209/92)	Cross-sectional	Firefighters	Before	Job satisfaction (SDQ), 719 (55.3)	9 – low risk
							Turnover intention (SDQ), 275 (21.1)	
Saukonautio et al. (2024) [35]	Finland, Europe	32.9 ± 7.3	n = 616, gender = 288/311	Cross-sectional	Mixed	During	Job satisfaction (EMS-RIS), 435 (70.6)	8 – moderate risk
							Turnover intention, (EMS-RIS) 109 (17.7)	
Zedini et al. (2016) [36]	Tunisia, Africa	41.0 ± 9.4	n = 337, gender = 114/223	Cross-sectional	Paramedics	During	Job satisfaction (SDQ), 118 (70.6)	8 – moderate risk
							Turnover intention (SDQ), 205 (60.8)	

DS – disaster supplement, EMTs – emergency medical technicians, EMS-RIS – emergency medical services role identity scale, EMS-SAQ – emergency medical services safety attitudes questionnaire, F – female, IQ – intention to leave questionnaire, JSQ – job satisfaction questionnaire, N – total sample size, NA – not available, MSQ – Minnesota satisfaction questionnaire, SI – single item questionnaire, SDQ – self-developed questionnaire, SSI – staff satisfaction index, SQ – satisfaction questionnaire

### Quality of included studies

The study quality of the included studies was examined using a Hoy’s Risk of Bias assessment tool for prevalence studies [37]. The tool consists of 10 domains that assess both the internal and external validity of observational studies. External validity is evaluated through items 1–4, which examine factors such as selection bias and non-response bias, while internal validity is assessed through items 5–10, covering measurement bias (items 5–9) and biases related to data analysis (item 10). The overall risk of bias is determined by summing the scores of all 10 items, with each item assigned a score of 1 for low risk and 0 for high risk. Studies with a total score of 9–10 are classified as having a low risk of bias, scores of 7–8 indicate a moderate risk, and scores ranging from 0–6 signify a high risk of bias. Any discrepancies between GH and KJB were resolved through discussions with a third expert reviewer (**Table 1**).

### Data synthesis and analysis

The Freeman-Tukey double-arcsine transformation model was used to estimate the pooled prevalence of job satisfaction and turnover intention among EMS personnel with the metaprop package version 8.0-2 in R software [38,39]. The Freeman-Tukey double-arcsine transformation model uses p_i_ as the proportion estimate from study *i* in the analysis (i = 1,…, N). The overall job satisfaction and turnover intention prevalence estimates p_i_, were calculated as pi = e_i_/n_i_, with e_i_ being the number of job satisfaction or turnover intention and n_i_ being the total sample size of EMS personnel in the included studies, respectively. The Freeman-Tukey double-arcsine transformation model also calculates weighted pooled estimates and then performs back-transformation on the pooled estimates to stabilise the within-study variance by using the binomial distribution. Furthermore, the statistical analysis employed the inverse variance method, t-distribution for prediction interval, and Clopper-Pearson confidence interval for individual studies. Publication bias was assessed through Peter’s Regression Test [40] and visual inspection of the funnel plot [41].

Heterogeneity assessment [38,39] accounted for statistical differences of job satisfaction and turnover intention prevalence estimates among the included studies. Heterogeneity was examined using the restricted maximum-likelihood method with restricted maximum-likelihood estimator for tau, Q-Profile method for confidence interval of tau^2^ and tau, Calculation of *I^2^* based on Cochran's Q statistic (*P* < 0.10). Heterogeneity was categorised with scores of 25% (low), 50% (moderate), and 75% (high), respectively.

### Moderator analysis

Moderator analysis was performed among the included studies using pre-specified individual and methodological factors [42]. Meta-regression was performed for age, while sub-group analysis was performed for categorical variables including:

(1) EMS personnel (paramedics, EMTs, firefighters, and mixed personnel),

(2) COVID-19 status (during and before),

(3) continent (Africa, Asia, Europe, and North America),

(4) country income status (high, upper-middle, and lower-middle),

(5) assessment tool (structured questionnaires and non-structured questionnaires),

(6) study design (cross-sectional and retrospective),

(7) study quality (low risk of bias and moderate risk of bias),

(8) sample size (<1000 and >1000) (**Table 2**).

**Table 2:** Moderator analysis for job satisfaction and turnover intention

Job satisfaction			Turnover intention		
**Characteristics**	**n**	**Prevalence (CI)**	**Characteristics**	**n**	**Prevalence (CI)**
Age (*P* = 0.231)	31	−0.008 (−0.022, 0.005)	Age (*P* = 0.881)	25	0.0008 (−0.010, 0.012)
EMS personnel (*P* = 0.478)			EMS personnel (*P* < 0.007)*		
*EMTs*	6	0.71 (0.37, 0.91)	*Paramedics*	11	0.28 (0.19, 0.39)
*Firefighters*	4	0.62 (0.47, 0.74)	*EMTs*	5	0.22 (0.13, 0.35)
*Paramedics*	11	0.54 (0.35, 0.72)	*Firefighters*	2	0.17 (0.11, 0.25)
*Mixed personnel*	10	0.70 (0.58, 0.80)	*Mixed personnel*	8	0.37 (0.28, 0.47)
COVID-19 status (*P* = 0.364)			COVID-19 status (*P* = 0.298)		
*During COVID-19*	4	0.56 (0.41, 0.70)	*During COVID-19*	5	0.34 (0.23, 0.48)
*Before COVID-19*	27	0.65 (0.53, 0.75)	*Before COVID-19*	20	0.27 (0.21, 0.34)
Continent (*P* < 0.0001)*			Continent (*P* < 0.0001)*		
*North America*	21	0.67 (0.53, 0.79)	*Africa*	1	0.61 (0.55, 0.66)
*Asia*	5	0.64 (0.54, 0.72)	*Europe*	4	0.37 (0.22, 0.55)
*Europe*	4	0.52 (0.32, 0.71)	*North America*	16	0.26 (0.20, 0.34)
*Africa*	1	0.35 (0.30, 0.40)	*Asia*	4	0.21 (0.17, 0.25)
Country income status (*P* < 0.0001)*			Country income status (*P* < 0.0001)*		
*High income*	27	0.64 (0.52, 0.75)	*High income*	24	0.27 (0.22, 0.33)
*Upper-middle income*	3	0.67 (0.54, 0.79)	*Upper-middle income*	-	-
*Low income*	1	0.35 (0.30, 0.40)	*Lower-middle income*	1	0.61 (0.55, 0.66)
Assessment tool (*P* = 0.158)			Assessment tool (*P* = 0.622)		
*Structured questionnaire*	17	0.70 (0.58, 0.80)	*Structured questionnaire*	13	0.27 (0.18, 0.38)
*Non-structured questionnaire*	14	0.55 (0.38, 0.71)	*Non-structured questionnaire*	12	0.30 (0.23, 0.37)
Study quality (*P* = 0.742)			Study quality (*P* < 0.046)*		
*Moderate risk*	22	0.65 (0.50, 0.77)	*Moderate risk*	17	0.25 (0.18, 0.39)
*Low risk*	9	0.62 (0.49, 0.73)	*Low risk*	8	0.37 (0.28, 0.46)
Study design (*P* = 0.202)			Study design (*P* = 0.033)		
*Retrospective*	7	0.75 (0.54, 0.79)	*Retrospective*	7	0.19 (0.12, 0.39)
*Cross-sectional*	24	0.60 (0.48, 0.71)	*Cross-sectional*	18	0.32 (0.26, 0.40)
Sample size (*P* = 0.769)			Sample size (*P* = 0.149)		
*<1000*	17	0.65 (0.51, 0.77)	*<1000*	10	0.24 (0.18, 0.30)
*>1000*	14	0.62 (0.45, 0.77)	*>1000*	15	0.32 (0.23, 0.42)

CI – confidence interval, EMS – emergency medical services, EMTs – emergency medical technicians, n – number of prevalence estimates

*Significant moderator variables.

## RESULTS

**Figure 1:**
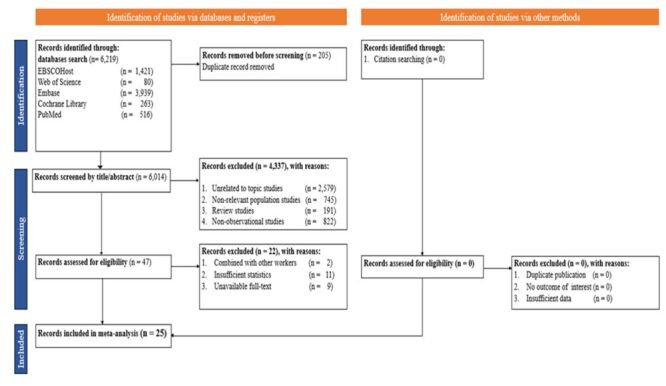
PRISMA flowchart for study selection.

We identified 6219 studies from electronic databases, Google searches, and reference lists, with 25 studies [13–36,43] published between 1993 and 2024 included in the analysis (**Figure 1**, **Table 1**). The sample sizes in the included studies ranged from 99 to 18 824, with a total of 59 562 EMS personnel. Mean age in the studies ranged from 30 to 41. Majority of the studies (n = 19) employed a cross-sectional design using survey-based methods while other studies (n = 6) employed retrospective designs using administrative or registry data sets. Some studies (n = 7) were conducted during the COVID-19 pandemic, while other studies (n = 18) were conducted in the pre-pandemic period. Most of the studies were conducted in the USA, with others in Germany, Finland, Israel, Saudi Arabia, Japan, South Korea, Malaysia, Tunisia, and Iran. Across the world, studies were conducted in Africa (n = 1), North America (n = 15), Europe (n = 4), and Asia (n = 5). Job satisfaction was assessed using the single-item (SI) questionnaire, satisfaction questionnaire (SQ), self-developed questionnaires (SDQ), Minnesota satisfaction questionnaire (MSQ), EMS role identity scale (EMS-RIS) and EMS safety attitudes questionnaire (EMS-SAQ), while turnover intention was measured using the intention to leave questionnaire (IQ), SI, SDQ, as well as the EMS-RIS. The overall quality of the included studies was rated as moderate risk of bias (n = 16) to low risk of bias (n = 9) (**Table 1**).

### Prevalence of job satisfaction among EMS personnel

**Figure 2:**
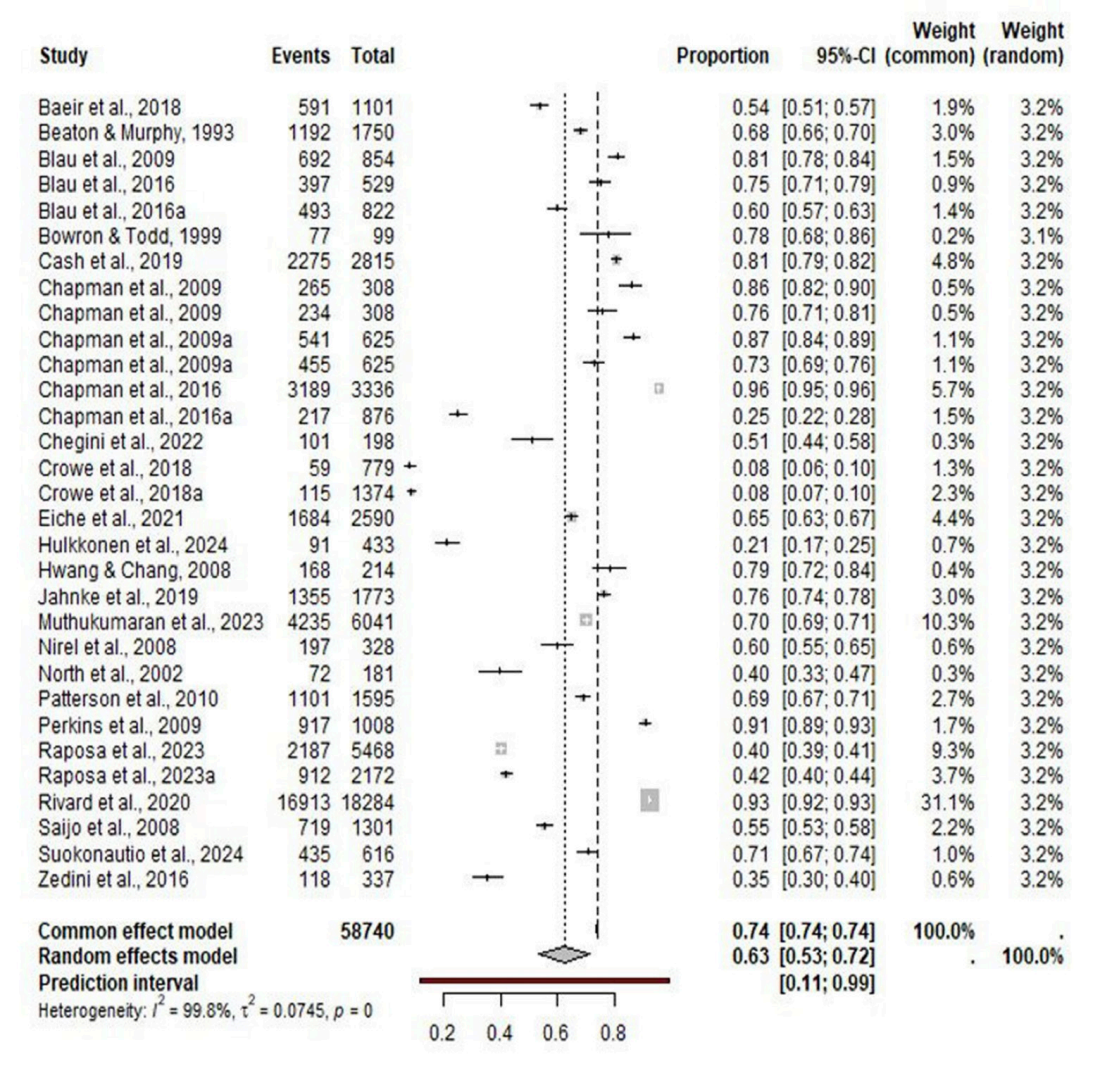
Prevalence of job satisfaction among emergency medical services personnel.

A total of 41 997 EMS personnel were satisfied with the emergency services job among the included studies. The pooled prevalence of job satisfaction was estimated at 63% (95% CI = 53%, 72%), suggesting that six out of ten EMS personnel are satisfied with the emergency services profession. Visual inspection of the funnel plot (Figure S1 in the **Online Supplementary Document**) and the results of Peter’s regression test did not suggest evidence of publication bias with an estimate of −39.3 (standard error (SE) = 45.9), *t* = −0.9, df = 31, and *P* = 0.40. The prediction interval for future prevalence studies was estimated to be between 11 and 99%. Among the included studies, statistical heterogeneity was observed, with Cochrane Q = 17 641.8, τ^2^ = 0.0745, and τ^2^ = 99.8% (*P* < 0.001) (**Figure 2**).

Across continents the prevalence rates were as follows: North America, 67% (95% CI = 53%, 79%); Asia, 64% (95% CI = 54%, 72%); Europe, 52% (95% CI = 32%, 71%); and Africa, 35% (95% CI = 30%, 40%). Among the countries, the prevalence rates were as follows: South Korea, 79% (95% CI = 73%, 84%); Malaysia, 70% (95% CI = 69%, 71%); the USA, 66% (95% CI = 52%, 78%); Israel, 60% (95% CI = 55%, 65%); Germany, 60% (95% CI = 52%, 67%); Japan, 55% (95% CI = 53%, 58%); Finland, 44% (95% CI = 15%, 79%); and Tunisia, 35% (95% CI = 30%, 40%) (**Table 2**).

### Results of the moderator analysis for job satisfaction

Age (*P* = 0.23) was associated with a non-significant decrease in job satisfaction with regression coefficient of -0.008 (95% CI = −0.022, 0.005). Among the types of EMS personnel (*P* = 0.49), the prevalence rate was 71% (95% CI = 37%, 91%) for EMTs, 62% (95% CI = 47%, 74%) for firefighters, 54% (95% CI = 35%, 72%) for paramedics, and 70% (95% CI = 58%, 80%) for mixed personnel. Regarding assessment tool (*P* = 0.16), the prevalence rate was 70% (95% CI = 58%, 80%) for structured questionnaires and 55% (95% CI = 38%, 71%) for non-structured questionnaires. Relating to the COVID-19 pandemic (*P* = 0.36), the prevalence rate was 56% (95% CI = 41%, 70%) during the pandemic and 65% (95% CI = 53%, 75%) in the pre-pandemic period. Regarding country income level (*P* < 0.0001), the prevalence rate was 67% (95% CI = 54%, 79%) for middle-income countries, 64% (95% CI = 52%, 75%) for high-income countries, and 35% (95% CI = 30%, 40%) for low-income countries. Regarding study quality (*P* = 0.74), the prevalence rate was 65% (95% CI = 50%, 77%) for moderate-risk studies and 62% (95% CI = 49%, 73%) for low-risk studies. Regarding study design (*P* = 0.20), the prevalence rate was 75% (95% CI = 54%, 89%) for retrospective studies and 60% (95% CI = 48%, 71%) for cross-sectional studies. Regarding sample size (*P* = 0.77), the prevalence rate was 65% (95% CI = 45%, 77%) for studies with fewer than 1000 participants and 62% (95% CI = 51%, 77%) for studies with more than 1000 participants (**Table 2**).

### Prevalence of turnover intention among EMS personnel

**Figure 3:**
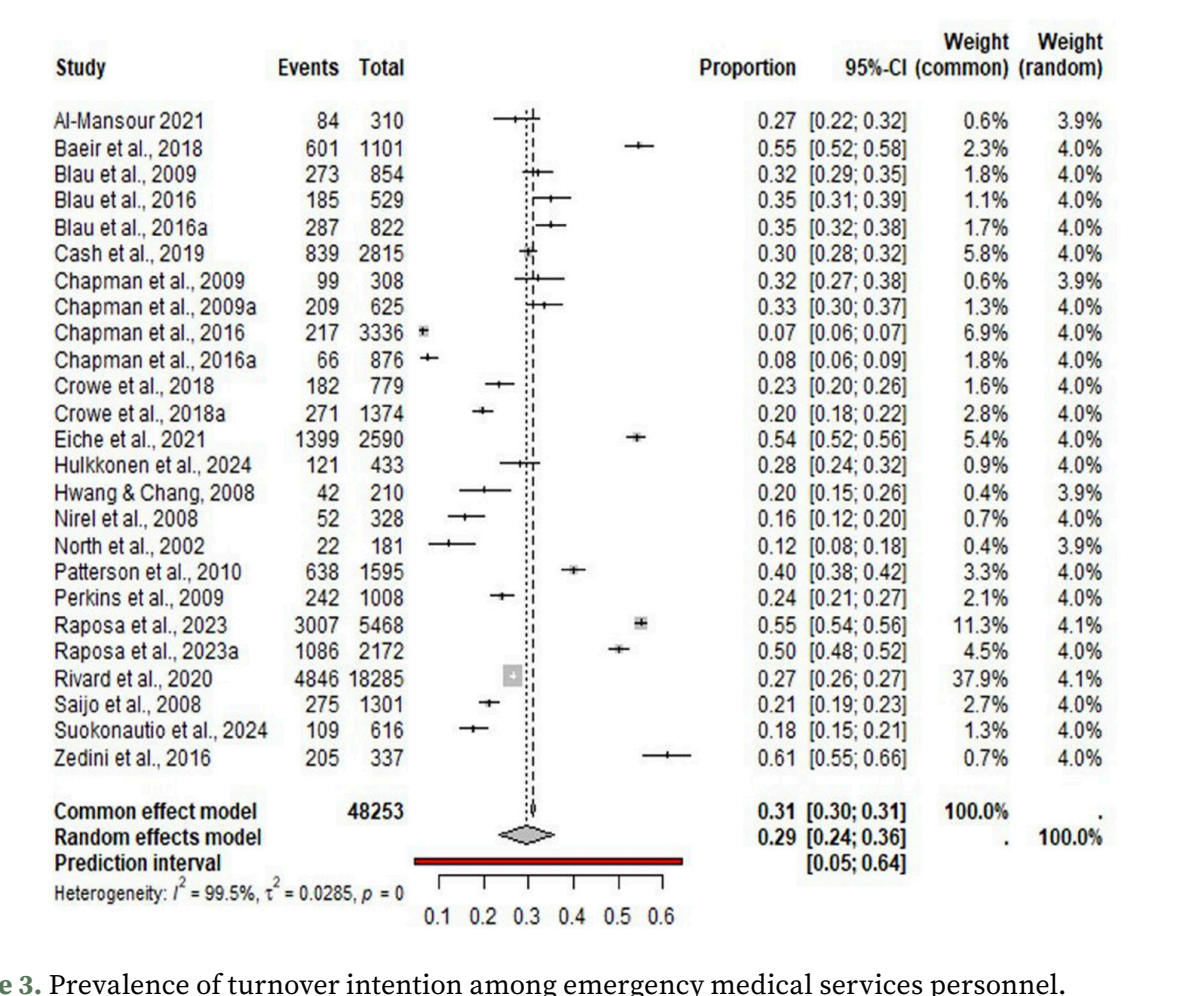
Prevalence of turnover intention among emergency medical services personnel.

A total of 15 357 EMS personnel intended to leave (turnover intention) the EMS profession among the included studies. The pooled prevalence of turnover intention was estimated at 29% (95% CI = 24%, 36%), indicating that three out of every ten EMS personnel intended to leave the EMS profession. Visual inspection of the funnel plot (Figure S2 in the **Online Supplementary Document**) and the results of Peter’s regression test did not suggest evidence of publication bias with an estimate of −10.4 (SE = 42.3), *t* = −0.25, df = 23, *P* = 0.81. The prediction interval for future prevalence studies was estimated to be between 5% and 64%. Among the included studies, statistical heterogeneity was observed, with Cochrane Q = 5024.3, τ^2^ = 0.0285, τ^2^ = 99.5% (*P* < 0.001) (**Figure 3**).

Across continents the prevalence rates were as follows: Africa, 61% (95% CI = 56%, 66%); Europe, 37% (95% CI = 22%, 55%); Asia, 26% (95% CI = 20%, 34%); and North America, 21% (95% CI = 17%, 25%). Among the countries, the prevalence rates were as follows: Tunisia, 61% (95% CI = 55%, 66%); Germany, 54% (95% CI = 53%, 56%); Saudi Arabia, 27% (95% CI = 22%, 32%); the USA, 26% (95% CI = 20%, 34%); Finland, 22% (95% CI = 16%, 30%); Japan, 21% (95% CI = 19%, 23%); South Korea, 20% (95% CI = 15%, 26%); and Israel, 16% (95% CI = 12%, 20%) (**Table 2**).

### Results of the moderator analysis for turnover intention

Age (*P* = 0.88) was associated with a non-significant increase in turnover intention with regression coefficient of 0.0008 (95% CI = −0.010, 0.012). Among the types of EMS personnel (*P* < 0.007), the prevalence of turnover intention was 28% (95% CI = 19%, 39%) for paramedics, 22% (95% CI = 13%, 35%) for EMTs, 17% (95% CI = 11%, 25%) for firefighters, and 37% (95% CI = 28%, 47%) for mixed EMS personnel. Regarding study quality (*P* < 0.05), the prevalence rates were 38% (95% CI = 29%, 46%) for low-risk of bias studies and 23% (95% CI = 17%, 30%) for moderate-risk of bias studies. Regarding study design (*P* < 0.03), the prevalence rates were 19% (95% CI = 12%, 29%) for retrospective studies and 32% (95% CI = 26%, 40%) for cross-sectional studies. Regarding assessment tool (*P* < 0.01), the prevalence rates were 40% (95% CI = 55%, 66%) for structured questionnaires and 25% (95% CI = 19%, 33%) for non-structured questionnaires. When comparing data relation to the COVID-19 pandemic (*P* = 0.30), turnover intention was 34% (95% CI = 23%, 48%) during the pandemic and 27% (95% CI = 21%, 34%) in the pre-pandemic period. Regarding country income level (*P* < 0.0001), the prevalence rates were 60% (95% CI = 55%, 66%) for lower-middle-income countries and 27% (95% CI = 22, 33%) for high-income countries. Regarding sample size (*P* = 0.15), the prevalence rates were 24% (95% CI = 18%, 30%) for studies with fewer than 1000 participants and 32% (95% CI = 23%, 42%) for studies with more than 1000 participants (**Table 2**).

## DISCUSSION

To our knowledge, this meta-analysis is the first to comprehensively estimate the prevalence of job satisfaction and turnover intention among EMS personnel, approximately six in ten EMS workers reported being satisfied with their jobs, while three in ten expressed an intention to leave the profession. Country income level and continent significantly influenced both job satisfaction and turnover intention, although limited number of studies demonstrated lack of statistical power. Study quality, study design, and assessment tools, also significantly affected turnover intention, demonstrating the influence of rigorous study methods and standardised instruments. However, age, COVID-19 status, and sample size were not significant moderators, which may reflect limited statistical power as well as unexplored organisational and health care system factors. Collectively, these findings highlight the complex interplay of occupational, contextual, and methodological factors that shape workforce experiences in EMS.

### Job satisfaction among EMS personnel

The findings revealed that more than half of EMS personnel are satisfied with their jobs, with job satisfaction being an important part of occupational well-being. EMS personnel are routinely exposed to physically and emotionally demanding situations [10,44]. EMS professionals frequently manage unpredictable high-acuity cases, make time-sensitive decisions, and work rotating shifts, often with limited institutional support. These stressors challenge both physical endurance and psychological resilience, with evidence showing that high workload, irregular schedules, and exposure to traumatic incidents predispose EMS personnel to burnout, posttraumatic stress disorder, and emotional exhaustion, negatively affecting job satisfaction [10]. Consistent these findings, Abd-Ellatif et al. (2021) [45] and Huang et al. (2022) [3] reported that EMS personnel faced heightened mental health risks, increased workloads, and concerns for personal safety during the COVID-19 pandemic. In our analysis, job satisfaction was lower during the pandemic (56%) compared with the pre-pandemic period (65%), although the difference was not statistically significant. These findings suggest that the cumulative nature of EMS work-related stressors may affect job satisfaction over time, especially in systems lacking adequate support mechanisms. As such, strengthening organisational support and providing opportunities for professional growth are therefore essential to enhance job satisfaction and ensure the sustainability of pre-hospital emergency services.

Moderator analyses provided additional insights into factors contributing to heterogeneity, although most did not reach statistical significance. Low risk of bias studies reported slightly lower job satisfaction compared with moderate risk studies, suggesting that more rigorous designs may produce conservative and reliable estimates. Similarly, structured questionnaires yielded higher prevalence than non-structured tools, indicating that assessment tools may still influence the prevalence estimates. Other moderators, including EMS personnel, study design, age, COVID-19 status, and sample size, did not show significant differences. The possible explanation for the lack of significant moderator effects may highlight reduced statistical power, particularly since some subgroup categories were represented by a single study. In addition, the findings suggest that unexplored contextual factors, such as organisational support, health care system capacity, and cultural attitudes toward EMS, may exert greater influence than individual- or study-level variables. Thus, these findings highlight the importance of reporting detailed individual, methodological, and organisational variables to better under contributing factors of heterogeneity.

Furthermore, the study also emphasises the challenges EMS personnel face in maintaining job satisfaction given the high-stakes and demanding nature of their work. Previous research has shown that dissatisfaction among emergency nurses and health professionals is often linked to burnout, critical decision-making pressures, fear of medical errors, and the burden of adverse patient outcomes [46]. These stressors are equally relevant for EMS personnel, whose work requires rapid responses under physically and psychologically taxing conditions with interventions that address role-specific challenges being critical to sustaining satisfaction. EMS personnel in high-income countries such as the USA and Canada may often benefit from better-resourced health care systems and stronger institutional support, whereas those in low- and middle-income countries face systemic challenges such as inadequate medical supplies, lower wages, insufficient training, and weak organisational support structures. Evidence from Gebrekidan et al. (2023) [47] and Mafula et al. (2025) [48] further indicates that the absence of career development opportunities undermines satisfaction among health care workers. Overall, while EMS personnel share common occupational stressors worldwide, improving job satisfaction requires interventions that strengthen organisational support, optimise resources, and promote professional development opportunities.

### Job satisfaction among EMS personnel

The study findings indicate that approximately three in ten EMS personnel expressed an intention to leave their jobs, highlighting a substantial risk of workforce instability within EMS profession. These findings are consistent with previous literature documenting high turnover intentions among health care professionals, where job-related stress, burnout, and demanding work environments are frequent contributors [49,50]. Ren et al. (2024) [51] reported that 45% of emergency nurses intended to leave their positions, with higher rates observed in Asia (54%) and among younger professionals under the age of 35 (61%). Similarly, Yan et al. (2021) [52] found that 50% of emergency physicians in China reported turnover intention, citing occupational stress, depression, poor sleep, shift work, and workplace violence as major predictors. High turnover in EMS is particularly concerning because it exacerbates staffing shortages, disrupts teamwork, and threatens the quality and continuity of pre-hospital emergency care. Previous studies also highlight low morale, job dissatisfaction, poor pay, and limited career development as important drivers of turnover [46,53–55]. These findings suggest that organisational and system-level interventions, including improving working conditions, supporting mental health, and expanding career opportunities, are needed to reduce turnover intention in EMS.

Turnover intention among EMS personnel varied across geographic, economic, and methodological contexts, reflecting the interplay of occupational stressors and health care system structures. Systemic underinvestment in health personnel, limited professional recognition, and persistent resource shortages, as highlighted by Gebrekidan et al. (2023) [47] and Mafula et al. (2025) [48], contribute to higher turnover rates in resource-constrained settings. Differences were also observed across EMS personnel, with paramedics reporting higher turnover intention than EMTs and firefighters, likely due to greater role complexity and exposure to critical stressors. Nevertheless, Roth et al. (2024) [55] and Heponiemi et al. (2024) [56] have shown that supportive workplace environments and comprehensive health care policies, such as those in Switzerland and Finland, can mitigate turnover risks. Moderator analyses further showed that study quality, study design, and measurement tools significantly influenced prevalence estimates. Higher turnover intention was reported in low-risk studies, cross-sectional designs, and those using structured questionnaires, suggesting that rigorous methodologies and standardised instruments may accurately capture workforce instability. However, age, COVID-19 status, and sample size were not significant moderators. While prevalence was slightly higher during the COVID-19 pandemic compared with pre-pandemic levels, suggesting both resilience among EMS personnel and variation in institutional responses during the crisis. Therefore, addressing both systemic and organisational determinants through improved health care financing, strengthened institutional support, and expanded professional development opportunities may help to reduce turnover intention and ensure a sustainable EMS workforce.

### Strength and limitations

The current meta-analysis offers valuable insights into the prevalence of job satisfaction and turnover intention among EMS personnel, presenting several study strengths. (i) This is the first meta-analysis of 25 studies with 59 562 EMS personnel to determine the prevalence of job satisfaction and turnover intention. (ii) The study followed strict methodological procedures by conducting comprehensive literature search and adherence to MOOSE guidelines ensuring high methodological rigor. (iii) The study used robust statistical methods, including sub-group analyses to identify significant moderator variables and publication bias to identify small-study effects. However, the meta-analysis is still limited by substantial heterogeneity among included studies, limited geographic representation predominantly from North America. While the study provides a rigorous examination of job satisfaction and turnover intention in EMS personnel, there is need for further research to explore across diverse geographic settings, especially in underrepresented regions, to develop effective job satisfaction and retention strategies.

## CONCLUSIONS

The study demonstrated that 63% of EMS workers reported job satisfaction, while 29% expressed an intention to leave the profession, underscoring a considerable risk of workforce instability within emergency medical services. This meta-analysis highlights the challenges of sustaining job satisfaction and reducing turnover intention among EMS personnel. Significant moderators, including country income level, continent, personnel type, study design, study quality, and measurement tools, suggested structural, organisational, and methodological factors influence on satisfaction and turnover intention. In contrast, age, COVID-19 status, and sample size were not significant, reflecting reduced statistical power due to limited number of studies. Moreover, these findings suggest that while EMS personnel face common occupational stressors worldwide, addressing these challenges will require improved organisational support, workload management, mental health resources, and professional development opportunities, especially in resource-limited settings. Future research employing standardised measurement instruments and longitudinal designs is warranted to clarify the dynamic interplay between job satisfaction and turnover intention and to identify priority areas for intervention.

## Additional material


Online Supplementary Document
