# Supplementary material for: Overview of the prevalence of job satisfaction and turnover intention among emergency medical services personnel: a meta-analysis
Source: J Glob Health. 2025 Nov 28;15:04320. doi: 10.7189/jogh.15.04320 (PMC12662026; doi:10.7189/jogh.15.04320)

Supplement to: Huang G, Hung WK, Ngolombe R, Maona C, Chiona BC, Banda KJ. Overview of the prevalence of job satisfaction and turnover intention among emergency medical services personnel: A meta-analysis. J Glob Health. 2025;15:04320.

Table S1. Search Strategy……………………………………………….…………….…………2

Figure S1. Funnel Plot for Publication Bis of Job Satisfaction……………………………….5

Figure S2: Funnel Plot for Publication Bis of Turnover Intention……………………………6

Table S1. Search Strategy

Database	Keywords	Number of studies
EBSCOHost	TX (prevalence OR incidence OR epidemiology OR rate OR rates OR number OR proportion OR probability OR event) AND TX (job satisfaction OR intrinsic satisfaction OR extrinsic satisfaction OR turnover intention OR intention to leave OR attrition) AND TX (Emergency Medical Services Personnel OR EMS personnel OR ambulance personnel OR fire fighters OR first responders OR paramedics OR emergency medical technicians OR EMTs) AND TX (observational studies OR cohort study OR cross-sectional study).<br>**Limiters** - Full Text<br>**Expanders** - Apply equivalent subjects<br>**Search modes** - Find all my search terms	1,421
Cochrane Library	prevalence OR incidence OR epidemiology OR rate OR rates OR number OR proportion OR probability OR event in Title Abstract Keyword AND job satisfaction OR intrinsic satisfaction OR extrinsic satisfaction OR turnover intention OR intention to leave OR attrition in Title Abstract Keyword AND Emergency Medical Services Personnel OR EMS personnel OR ambulance personnel OR fire fighters OR first responders OR paramedics OR emergency medical technicians OR EMTs in Title Abstract Keyword - (Word variations have been searched)	263
PubMed	("epidemiology"[MeSH Subheading] OR "epidemiology"[All Fields] OR "prevalence"[All Fields] OR "prevalence"[MeSH Terms] OR "prevalance"[All Fields] OR "prevalences"[All Fields] OR "prevalence s"[All Fields] OR "prevalent"[All Fields] OR "prevalently"[All Fields] OR "prevalents"[All Fields] OR ("epidemiology"[MeSH Subheading] OR "epidemiology"[All Fields] OR "incidence"[All Fields] OR "incidence"[MeSH Terms] OR "incidences"[All Fields] OR "incident"[All Fields] OR "incidents"[All Fields]) OR ("epidemiologies"[All Fields] OR "epidemiology"[MeSH Subheading] OR "epidemiology"[All Fields] OR "epidemiology"[MeSH Terms] OR "epidemiology s"[All Fields]) OR ("j rehabil assist technol eng"[Journal] OR "rate"[All Fields]) OR "rates"[All Fields] OR ("number"[All Fields] OR "numbers"[All Fields]) OR ("proportion"[All Fields] OR "proportions"[All Fields]) OR ("probability"[MeSH Terms] OR "probability"[All Fields] OR "probabilities"[All Fields]) OR ("event"[All Fields] OR "event s"[All Fields] OR "events"[All Fields])) AND ("job satisfaction"[MeSH Terms] OR ("job"[All Fields] AND "satisfaction"[All Fields]) OR "job satisfaction"[All Fields] OR (("intrinsic"[All Fields] OR "intrinsically"[All Fields] OR "intrinsics"[All Fields]) AND ("personal satisfaction"[MeSH Terms] OR ("personal"[All Fields] AND "satisfaction"[All Fields]) OR "personal satisfaction"[All Fields] OR "satisfaction"[All Fields] OR "satisfactions"[All Fields] OR "satisfaction s"[All Fields])) OR (("extrinsic"[All Fields] OR "extrinsically"[All Fields] OR "extrinsics"[All Fields]) AND ("personal satisfaction"[MeSH Terms] OR ("personal"[All Fields]	516

	AND "satisfaction"[All Fields]) OR "personal satisfaction"[All Fields] OR "satisfaction"[All Fields] OR "satisfactions"[All Fields] OR "satisfaction s"[All Fields])) OR (("personnel turnover"[MeSH Terms] OR ("personnel"[All Fields] AND "turnover"[All Fields]) OR "personnel turnover"[All Fields] OR "turnovers"[All Fields] OR "turnover"[All Fields]) AND ("intention"[MeSH Terms] OR "intention"[All Fields] OR "intent"[All Fields] OR "intentions"[All Fields] OR "intentional"[All Fields] OR "intentioned"[All Fields] OR "intents"[All Fields])) OR (("intention"[MeSH Terms] OR "intention"[All Fields] OR "intent"[All Fields] OR "intentions"[All Fields] OR "intentional"[All Fields] OR "intentioned"[All Fields] OR "intents"[All Fields]) AND ("leave"[All Fields] OR "leaved"[All Fields] OR "leaving"[All Fields] OR "plant leaves"[MeSH Terms] OR ("plant"[All Fields] AND "leaves"[All Fields]) OR "plant leaves"[All Fields] OR "leaves"[All Fields])) OR ("attritions"[All Fields] OR "tooth attrition"[MeSH Terms] OR ("tooth"[All Fields] AND "attrition"[All Fields]) OR "tooth attrition"[All Fields] OR "attrition"[All Fields])) AND ((("emergency medical services"[MeSH Terms] OR ("emergency"[All Fields] AND "medical"[All Fields] AND "services"[All Fields]) OR "emergency medical services"[All Fields]) AND ("occupational groups"[MeSH Terms] OR ("occupational"[All Fields] AND "groups"[All Fields]) OR "occupational groups"[All Fields] OR "personnel"[All Fields] OR "personnel s"[All Fields] OR "personnels"[All Fields])) OR (("emerg med serv"[Journal] OR "ems mag"[Journal] OR "ems"[All Fields]) AND ("occupational groups"[MeSH Terms] OR ("occupational"[All Fields] AND "groups"[All Fields]) OR "occupational groups"[All Fields] OR "personnel"[All Fields] OR "personnel s"[All Fields] OR "personnels"[All Fields])) OR (("ambulance s"[All Fields] OR "ambulances"[MeSH Terms] OR "ambulances"[All Fields] OR "ambulance"[All Fields]) AND ("occupational groups"[MeSH Terms] OR ("occupational"[All Fields] AND "groups"[All Fields]) OR "occupational groups"[All Fields] OR "personnel"[All Fields] OR "personnel s"[All Fields] OR "personnels"[All Fields])) OR ("firefighters"[MeSH Terms] OR "firefighters"[All Fields] OR ("fire"[All Fields] AND "fighters"[All Fields]) OR "fire fighters"[All Fields]) OR ("emergency responders"[MeSH Terms] OR ("emergency"[All Fields] AND "responders"[All Fields]) OR "emergency responders"[All Fields] OR ("first"[All Fields] AND "responders"[All Fields]) OR "first responders"[All Fields]) OR ("paramedic s"[All Fields] OR "paramedical"[All Fields] OR "paramedicals"[All Fields] OR "paramedics"[MeSH Terms] OR "paramedics"[All Fields] OR "paramedic"[All Fields] OR "emergency medical technicians"[MeSH Terms] OR ("emergency"[All Fields] AND "medical"[All Fields] AND "technicians"[All Fields]) OR "emergency medical technicians"[All Fields]) OR ("emergency medical technicians"[MeSH Terms] OR ("emergency"[All Fields] AND "medical"[All Fields] AND "technicians"[All Fields]) OR "emergency medical technicians"[All Fields]) OR "EMTs"[All Fields])	

Embase	1. 'prevalence'/exp OR prevalence OR 'incidence'/exp OR incidence OR 'epidemiology'/exp OR epidemiology OR rate OR rates OR 'number'/exp OR number OR proportion OR 'probability'/exp OR probability OR event<br>2. 'job satisfaction'/exp OR 'job satisfaction' OR (('job'/exp OR job) AND ('satisfaction'/exp OR satisfaction)) OR 'intrinsic satisfaction' OR (intrinsic AND ('satisfaction'/exp OR satisfaction)) OR 'extrinsic satisfaction' OR (extrinsic AND ('satisfaction'/exp OR satisfaction)) OR 'turnover intention'/exp OR 'turnover intention' OR (('turnover'/exp OR turnover) AND ('intention'/exp OR intention)) OR 'intention to leave' OR (('intention'/exp OR intention) AND to AND leave) OR 'attrition'/exp OR attrition<br>3. 'emergency medical services personnel' OR (('emergency'/exp OR emergency) AND medical AND services AND ('personnel'/exp OR personnel)) OR 'ems personnel'/exp OR 'ems personnel' OR (ems AND ('personnel'/exp OR personnel)) OR 'ambulance personnel'/exp OR 'ambulance personnel' OR (('ambulance'/exp OR ambulance) AND ('personnel'/exp OR personnel)) OR 'fire fighters'/exp OR 'fire fighters' OR (('fire'/exp OR fire) AND fighters) OR 'first responders'/exp OR 'first responders' OR (first AND responders) OR 'paramedics'/exp OR paramedics OR 'emergency medical technicians'/exp OR 'emergency medical technicians' OR (('emergency'/exp OR emergency) AND medical AND technicians) OR emts<br>4. 'observational studies'/exp OR 'observational studies' OR (observational AND ('studies'/exp OR studies)) OR 'cohort study'/exp OR 'cohort study' OR (('cohort'/exp OR cohort) AND ('study'/exp OR study)) OR 'cross-sectional study'/exp OR 'cross-sectional study' OR ('cross sectional' AND ('study'/exp OR study))<br>5.#1 AND #2 AND #3 AND #4	3,939
Web of Science	(prevalence OR incidence OR epidemiology OR rate OR rates OR number OR proportion OR probability OR event (All Fields) AND job satisfaction OR intrinsic satisfaction OR extrinsic satisfaction OR turnover intention OR intention to leave OR attrition (All Fields) AND Emergency Medical Services Personnel OR EMS personnel OR ambulance personnel OR fire fighters OR first responders OR paramedics OR emergency medical technicians OR EMTs (All Fields) AND observational studies OR cohort study OR cross-sectional study (All Fields)<br><br>https://www.webofscience.com/wos/woscc/summary/4c0f0d1d-0e8b-4642-b38d-a934018a0daf-0119e6425f/relevance/1	80

Figure S1: Funnel Plot for Publication Bis of Job Satisfaction

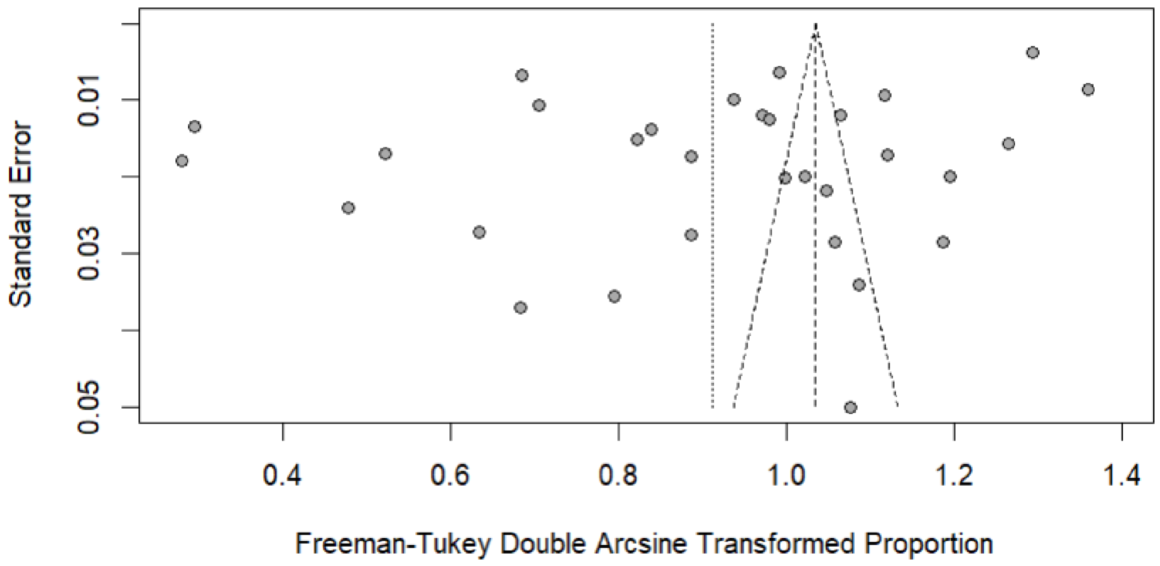

Figure S2: Funnel Plot for Publication Bis of Turnover Intent

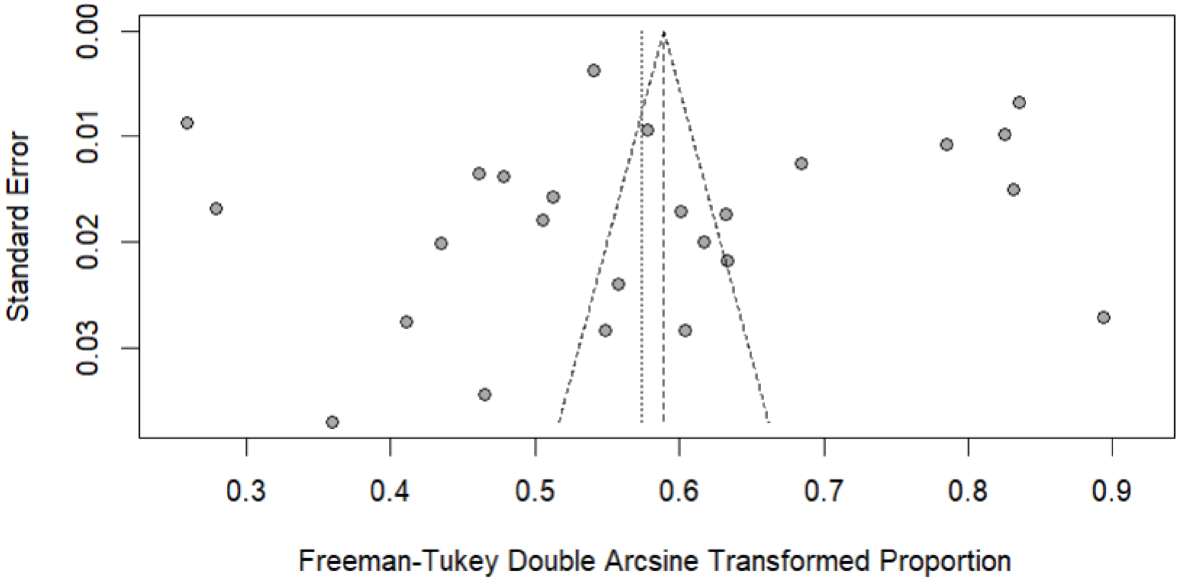

Supplement: Online Supplementary Document [file jogh-15-04320-s001.pdf]
